# Indoor Dust as a Source of Virulent Strains of the Agents of Cryptococcosis in the Rio Negro Micro-Region of the Brazilian Amazon

**DOI:** 10.3390/microorganisms8050682

**Published:** 2020-05-07

**Authors:** Fábio Brito-Santos, Luciana Trilles, Carolina Firacative, Bodo Wanke, Filipe Anibal Carvalho-Costa, Marília Martins Nishikawa, Jonas Pereira Campos, Angela Cristina Veríssimo Junqueira, Amanda Coutinho de Souza, Márcia dos Santos Lazéra, Wieland Meyer

**Affiliations:** 1 Mycology Laboratory, Evandro Chagas National Institute of Infectious Diseases, FIOCRUZ, Rio de Janeiro 21040-900, Brazilluciana.trilles@ini.fiocruz.br (L.T.); bodo.wanke@ini.fiocruz.br (B.W.); jonasc.rj@hotmail.com (J.P.C.); marcia.lazera@ini.fiocruz.br (M.S.L.); 2 Molecular Mycology Research Laboratory, Centre for Infectious Diseases and Microbiology, Faculty of Medicine and Health, Sydney Medical School, Westmead Clinical School, Marie Bashir Institute for Infectious Diseases and Biosecurity, The University of Sydney, Westmead Hospital (Research and Education Network), Westmead Institute for Medical Research, Sydney 2006, NSW, Australia; cfiracative@gmail.com; 3 Studies in Translational Microbiology and Emerging Diseases (MICROS) Research Group, School of Medicine and Health Sciences, Universidad del Rosario, Bogotá 541038, Colombia; 4 Laboratory of Molecular Epidemiology and Systematics, Oswaldo Cruz Institute, FIOCRUZ, Rio de Janeiro 21040-900, Brazil; carvalhocosta70@hotmail.com; 5 National Institute for Quality Control in Health, INCQS/FIOCRUZ, Rio de Janeiro 21040-900, Brazil; marilia.martins@incqs.fiocruz.br; 6 Laboratory of Parasitology, Oswaldo Cruz Institute, Rio de Janeiro 21040-900, Brazil; junqueir.rlk@terra.com.br (A.C.V.J.);

**Keywords:** *Cryptococcus neoformans*, *Cryptococcus gattii*, indoor dust, MLST, virulence, Brazilian Amazon

## Abstract

Cryptococcosis, a potentially fatal mycosis in humans, is acquired via exposure to exogenous environmental sources. This study aimed to investigate the frequency, genetic diversity, and virulence of cryptococcal strains isolated from indoor dust in the Rio Negro micro-region of the Brazilian Amazon. A total of 8.9% of the studied houses were positive, recovering nine *Cryptococcus neoformans* VNI and 16 *C. gattii* VGII isolates, revealing an endemic pattern in domestic microenvironments. The International Society for Human and Animal Mycology (ISHAM) consensus multilocus sequence typing (MLST) scheme for the *C. neoformans/C. gattii* species complexes identified two sequence types (STs), ST93 and ST5, amongst *C. neoformans* isolates and six STs amongst *C. gattii* isolates, including the Vancouver Island Outbreak ST7 (VGIIa) and ST20 (VGIIb), the Australian ST5, and ST264, ST268 and ST445, being unique to the studied region. Virulence studies in the *Galleria mellonella* model showed that five *C.* *gattii* strains and one *C. neoformans* strain showed a similar pathogenic potential to the highly virulent Vancouver Island outbreak strain CDR265 (VGIIa). The findings of this study indicate that humans can be exposed to the agents of cryptococcosis via house dust, forming the basis for future studies to analyze the impact of early and continuous exposure to indoor dust on the development of subclinical or clinical infections.

## 1. Introduction

Cryptococcosis is a potentially fatal respiratory and neurological mycosis affecting humans and animals worldwide. The disease is caused by two pathogenic members of the genus *Cryptococcus*, the *Cryptococcus neoformans* species complex and the *C. gattii* species complex [1]. Cryptococcosis caused by *C. neoformans* is cosmopolitan, affecting mainly immunocompromised individuals, especially HIV-infected patients, with an estimation of 223,000 new cases of cryptococcal meningitis alone each year in this group of patients [2]. On the other hand, *C. gattii,* which was previously associated with tropical and subtropical climates, predominantly causes a primary infection in immunocompetent individuals [3]. It is worthy of notice that endemic cryptococcosis by *C. gattii* shows a regional pattern in Brazil, being mostly reported in the north and northeast of the country, where it occurs usually in immunocompetent hosts, including children and young adults [4]. In 1999, cases of cryptococcosis by *C. gattii* appeared in British Columbia, Canada; at first, cases appeared in the form of outbreak related strains, later becoming endemic and persisting until today. Until 2015, 393 cases of cryptococcosis were reported in Canada, and currently Vancouver Island has one of the highest annual incidences of *C. gattii* infections amongst humans and animals in the world, making this region an important temperate endemic area of cryptococcosis [5].

Cryptococcosis is acquired by the inhalation of infectious propagules (desiccated yeasts cells or basidiospores) from the environment [6]. As such, the search for the ecological niche of the agents of cryptococcosis is a challenge, even though some studies have shown the presence of these yeasts in different environmental sources [7]. However, while *C. neoformans* can readily be isolated from pigeon guano and has been shown to grow and mate on mediums containing this substrate [8], *C. gattii* has not yet been isolated from pigeon excreta, but as has *C. neoformans,* it has instead been recovered from various tree species [9].

Pioneering studies on cryptococcosis in AIDS patients in Central Africa and Brazil demonstrated the risk of these patients acquiring cryptococcosis from indoor dust [10,11]. In Brazil, the first study describing the presence of *C. gattii* in dwellings in the hinterland of the Brazilian Amazon identified the genotypes ST7/VGIIb and ST20/VGIIa [12], which have been involved as causative agents in outbreaks elsewhere [5]. However, the residents of Santa Isabel do Rio Negro, a city in the hinterland of the Brazilian Amazon, who live in wooden houses might be exposed daily to the agents of cryptococcosis, which could also be happening in other cities with similar living styles in the Amazon region [12].

The present study in the Rio Negro micro-region, which is composed of four municipalities in the Amazon in Brazil, aimed to analyze the frequency, genetic diversity and virulence traits of the agents of cryptococcosis isolated from indoor dust.

## 2. Materials and Methods

### 2.1. Studied Region

**Figure 1:**
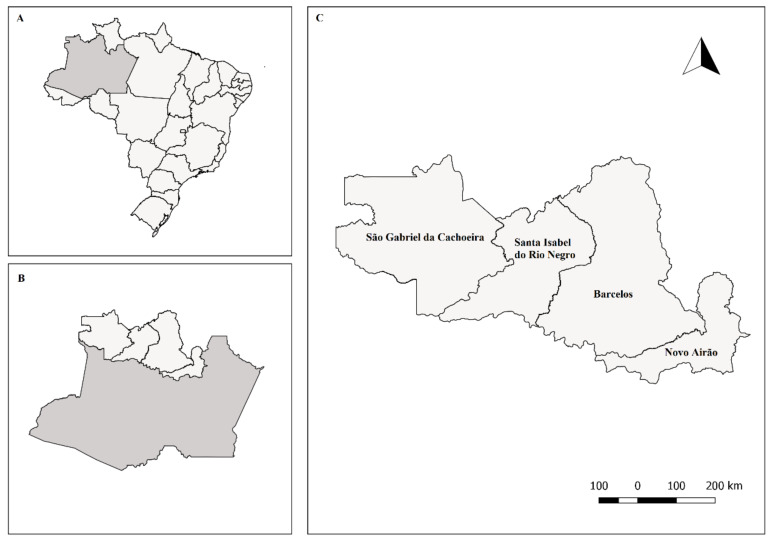
Location of the Amazonas state in Brazil (**A**) and location of the Rio Negro micro-region of the Brazilian Amazon (**B**), which is composed of four cities (**C**).

The study was conducted in the Amazonas state (Figure 1A) in the Rio Negro micro-region (Figure 1B), which is composed of four municipalities: Barcelos, Novo Airão, Santa Isabel do Rio Negro and São Gabriel da Cachoeira (Figure 1C). The Rio Negro, the largest left tributary of the Amazon River and the largest blackwater river in the world, runs across all those cities on the way to the capital of the Amazonas state, Manaus. This micro-region has an area of 332,278.183 km² and a combined population of 110,602 inhabitants according to the last Brazilian census. Three cities out of the four municipalities were included in this study: Santa Isabel, Barcelos and Novo Airão. The area encompasses the tropical rainforest climate, with a maximum temperature of 32.6 °C and a minimum temperature of 21.5 °C, and it is located at 21–40 m above the sea level. The cities’ settlement occurred approximately one hundred years ago, and the majority of the population lives in houses of wood or wood with masonry (Source: IBGE 2017).

### 2.2. Sampling and Isolation of Cryptococcal Strains

Indoor dust was collected from houses in the three municipalities in different neighborhoods of each city to investigate the presence of *C. neoformans* and *C. gattii*. After sweeping the house with a broom from each residence, one indoor dust sample per household was obtained. The study had the approval of each city’s Health Department, as well as ethical approval by the National Institute of Infectious Diseases Ethical Research Committee, Rio de Janeiro, Brazil (Reference No. CAAE 23238913.3.0000.5262). Seventy-nine samples of indoor dust were obtained from the three cities of the Rio Negro micro-region: 51 from Santa Isabel do Rio Negro, 12 from Barcelos and 16 from Novo Airão. Cryptococcal isolates were recovered as described previously [10,13]. Briefly, 1 g of each dust sample was suspended in 50 mL NaCl 0.9% with 0.2 g of chloramphenicol, followed by manual shaking for 5 min. After resting for 30 min, 1 mL of the supernatant was plated onto 10 Niger seed agar (NSA) plates (0.1 mL each). The plates were then incubated at 25 °C and checked daily for 5 days for growth of brown colonies. Phenol oxidase-positive or brown colonies were sub-cultured for phenotypic and molecular identification. The limit for the detection of phenol oxidase-positive colonies was 50 CFU per gram of sample.

### 2.3. Phenotypic Identification

Brown colonies were recovered and tested for urease production on Christensen urea agar and for carbon and nitrogen compound assimilation using VITEK 2-BioMerieux System (VITEK 2, ICB, bioMerieux, Durham, USA). The species *C. neoformans* and *C. gattii* were distinguished on canavanine-glycine-bromothymol blue (CGB) medium [12].

### 2.4. Molecular Characterization

After DNA extraction [14], the mating type was determined by PCR using specific primers for the pheromone genes as described previously [15]. Genotyping was performed according to the International Society for Human and Animal Mycology (ISHAM) consensus multilocus sequence typing (MLST) scheme for the *C. neoformans*/*C. gattii* species complexes, including the following seven unlinked genetic loc: *CAP59*, *GPD1*, *LAC1*, *PLB1*, *SOD1*, *URA5* and the IGS1 region [16]. The sequences were manually edited using the software Sequencher 5.3 (Gene Codes Corporation, MI, USA), and the allele types (AT) and the combined sequence types (STs) were identified via the MLST webpage [17]. All new STs were deposited on the online MLST database.

### 2.5. Phylogenetic Analyses

The genetic relationships of the 7 concatenate MLST loci were shown using the software Splitstree4 v. 4.14.5 [18]. Unrooted phylogenetic network analysis using the neighbor-net algorithm was performed for comparison of the sequence types (STs) identified in this study with 42 representative STs identified in different countries previously published [19].

### 2.6. Galleria mellonella Model

Larvae of the moth *G. mellonella* were used to evaluate the virulence of selected cryptococcal isolates recovered in this study. Larvae were obtained after the oviposition of adult moths reared and preserved at 26 °C and 60% relative humidity in the insectarium of the Westmead Hospital Animal Care Facility, Sydney, Australia. Ten larvae of similar size (about 3 g each) were selected, placed in a 90 mm plastic Petri dish and weighed before inoculation. Each fungal strain was grown on Sabouraud agar for 48 h at 27 °C. After cell counting using a Neubauer Chamber, an inoculum of 10^8^ yeast cells/mL was prepared in phosphate buffered saline (PBS), from which 10 µL were inoculated into the hemocoel of each larva by injection into the last left pro-leg, using a 50U insulin syringe with a 29-gauge needle. To monitor potential effects on survival due to physical injury, a group of 10 larvae was also inoculated with PBS, while another 10 larvae were not inoculated as a non-infected control. One group of larvae, inoculated with the well-characterized highly virulent strain *C. gattii* CDCR265, was included as a reference to determine the degree of virulence of the isolates. After injection, the larvae were incubated in Petri dishes at 37 °C for 10 days and checked daily for any mortality [20].

### 2.7. Statistical Analysis

Survival curves were graphed, median survival times were calculated and the estimation of differences in survival was analyzed by the Log-rank (Mantel-Cox) test (CI 95%) per strain. Median survival times were not determined (ND) when more than five larvae (50%) were alive at the end of the experiment. Statistical analysis was performed using GraphPad Prism version 7.00 for Windows (GraphPad Software, La Jolla, California, USA).

## 3. Results

From the 79 indoor dust samples taken, seven (8.9%) were positive for the agents of cryptococcosis: 3 from Santa Isabel do Rio Negro, 2 from Barcelos and 2 from Novo Airão (Table 1).

From the seven positive samples, nine *C. neoformans* VNI and 16 *C. gattii* VGII isolates were recovered and genotyped using the ISHAM consensus MLST scheme for the *C. neoformans/C. gattii* species complexes [16]. Amongst the 9 *C. neoformans* VNI isolates, ST93 (6/9) and ST5 (3/9) were identified, showing a clonal population. These two STs have previously been recovered worldwide. The most common *C. gattii* sequence type was ST7 (5 out of 16) (Vancouver Island Outbreak subtype VGIIb) and ST20 (2 out of 16) (Vancouver Island Outbreak subtype VGIIa). In addition, ST5 (1/16); previously described from Australia, and ST264 (2/16); ST265 (1/16); ST266 (1/16); ST267 (1/16); ST268 (1/16) and ST445 (2/16), unique STs to this region, were also identified.

**Figure 2:**
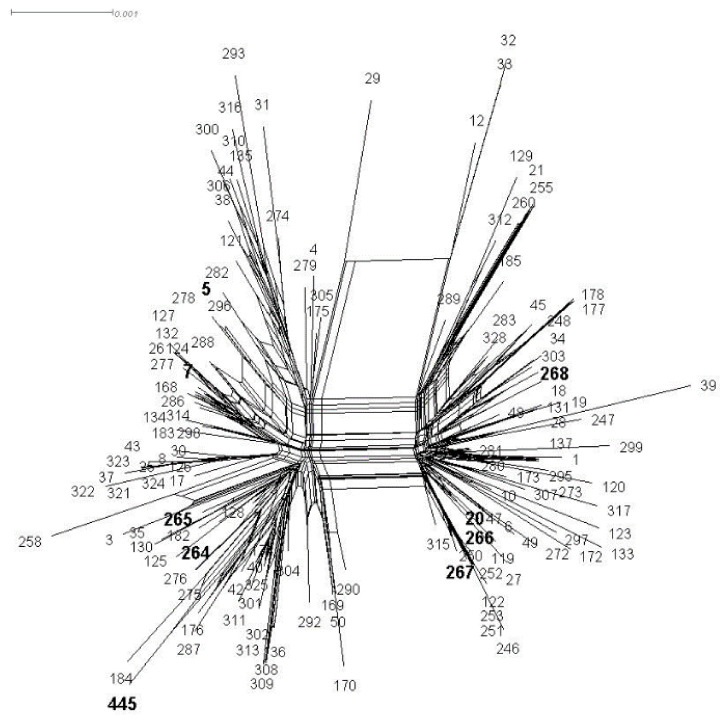
SplitsTree analysis of the combined multilocus sequence typing (MLST) loci showing the placement of the sequence types (STs) (numbers in bold) from the Rio Negro micro-region of the Brazilian Amazon, in context with the sequence types obtained from the global *C. gattii* VGII population (published data obtained from the website at mlst.mycologylab.org).

The analysis of the combined MLST loci showing the placement of the 16 isolated strains from the Rio Negro micro-region in the context of the STs obtained from the global *C. gattii* VGII population, which identifies the genetic diversity in this Amazon region, is shown in Figure 2. Mating type analysis demonstrated that most isolates are *MATα.* Only one *MAT*a isolate amongst the *C. gattii* isolates was found, strain SI443-17 (ST268).

In general, the average death rate of the *G. mellonella* larvae infected with *C. neoformans* VNI environmental isolates was slightly higher compared to that of the larvae infected with the *C. gattii* VGII environmental isolates, although without a statistical difference between the survival curves of the infected larvae (*p*-value = 0.6764). Amongst the VGII isolates, however, five isolates were of comparable virulence (*p*-value >0.05) to that of the Vancouver Island outbreak strain CDCR265. One isolate of ST7 (VGIIb), both isolates of ST20 (VGIIa) and the isolates with ST266 and ST267 demonstrated high virulence *in vivo* (Table 2). From the *C. neoformans* isolates, three (BAR10-16, BAR10-19, BAR08-1) belonging to the ST93 were as virulent as the VGIIa highly virulent Vancouver Island outbreak strain CDCR265 (*p* > 0.05) (Table 2).

## 4. Discussion

Cryptococcal infection is acquired through human and animal exposure to exogenous sources, as such the understanding of the dynamics and adaptation of these environmental reservoirs is of fundamental interest to seek answers to two main questions: 1. How do humans get infected? and 2. What are the means to avoid or reduce risks of infection? Humans are primarily exposed to organic indoor air inhalation, and as such, indoor dust is an important mechanism of exposure, as people spend over 86.9% of their lives in indoor environments [21]. Studies have found high levels of emerging contaminants in indoor dust worldwide, but those studies usually identify the fungal agent at genus level, missing the opportunity to detect pathogenic species/strains [22,23,24].

Studies on emerging contaminants in indoor dust and the resulting levels of human exposure to pathogenic *Cryptococcus* species/strains are scarce. Pioneering studies in Central Africa in the late 1980s detected a large number of *C. neoformans-*positive indoor dust samples in households of patients with AIDS-associated cryptococcosis [11,25]. Another similar study was carried out in the city of Rio de Janeiro, Brazil, by Passoni et al. (1998). The authors analyzed households of AIDS patients from the metropolitan area and found 13% positivity for *C. neoformans*. In addition, the authors observed that cryptococcosis was twice more frequent among AIDS patients residing in positive dwellings, thus suggesting an important role of positive indoor dust samples in the acquisition of cryptococcal infection in HIV/AIDS patients [10].

An initial local study in one municipality of the Rio Negro micro-region (Amazonas state, Brazil) revealed *C. gattii* isolates in indoor dust associated with wooden houses [12], suggesting the possibility of cryptococcal infection by *C. gattii* acquired from the domestic environment. In the present study, a larger area including two more adjacent cities (Barcelos and Novo Airão) in the same Amazon region (Figure 1) showed that 8.9% of the studied houses were positive for *C. gattii* and *C. neoformans*, revealing an endemic pattern and adaptation to domestic microenvironments in this region of Amazonia.

An indoor microbial study revealed special concerns for vulnerable groups, such as children, for the risk of indoor-acquired infection [26]. In Brazil, cryptococcosis by *C. gattii* manifesting as CNS infections in immunocompetent young adults and children of both sexes in the Amazon and the northeast regions of Brazil is common, with the associated lethality ranging from 35% to 40% [27,28,29,30]. The high incidence of *C. gattii* in indoor dust in the Rio Negro micro-region could explain the endemic pattern of cryptococcosis by VGII in children and young adults and thus indicates the need for future studies in other endemic areas to understand the role of the agents of cryptococcosis in indoor environments.

The molecular subtypes of cryptococcosis responsible for the epidemic in Canada were first described in 1999 (ST20, ST7), but they have since become endemic in the region. In subsequent studies, the same subtypes were identified in the Amazon region in different cities and at different time points [19]. MLST and whole genome sequencing studies suggest that possible multiple introductions of these subtypes have occurred into the North American Pacific Northwest and other parts of the world from South America [31]. ST7 (VGIIb) has been found all over the world, but ST20 (VGIIa), endemic in the Amazon region, was also found in the Rio Negro micro-region along with other new VGII STs, which are specific to this region. The genetic diversity of VGII in house dust correlates with the very diverse *C. gattii* VGII Brazilian population previously described and strongly supports the emergence of virulent strains from ancestors in the northern region of Brazil [19].

The globally most common *C. neoformans* genotypes, ST93 and ST5, were also identified in the Rio Negro micro-region. ST93 has been associated with infections in individuals infected with HIV in the Amazonia state and comprises the majority of clinical isolates in Southeastern Brazil [32]. ST5 is one of the most prevalent STs amongst the clinical isolates from Europe and Asia but was only rarely identified in clinical and environmental isolates from Brazil [33].

During the present study, there was no evidence of cryptococcosis cases in the inhabitants of the positive dwellings. However, the C*. neoformans* and *C. gattii* strains from the indoor dust analyzed in the present study were virulent when inoculated in the *G. mellonella* model. Comparison of the degree of virulence using the *G. mellonella* model showed that five genotypes of *C. gattii* VGII (ST7, ST20, ST266, ST267 and ST445) and one genotype of *C. neoformans* VNI (ST93) presented a similar pathogenic potential to that of the highly virulent Vancouver Island outbreak strain CDR265 (VGIIa, ST20) [34].

Microbiome studies of indoor dust and outdoor air samples in Boston and California (USA) detected a moderate percentage of the genus *Cryptococcus*, being the third most abundant genus in such samples [23,35]. These authors showed that the great majority of *Cryptococcus* spp. are non-pathogenic to humans. The present study showed that pathogenic *Cryptococcus* strains can also be found in indoor dust, posing a risk of cryptococcal infection for the inhabitants of the dwellings. The herein obtained findings reinforce the interplay of pathogenic fungal disease agents in the indoor environment as a possible source of infections of humans and animals, showing the importance of the application of the one health concept as an approach to understanding and counteracting fungal infections, especially in the immunocompromised (e.g., HIV-positive patients) or other vulnerable populations (children and the elderly), by assessing the indoor environmental health situation of those individuals to prevent potentially life-threatening fungal infections.

## 5. Conclusions

In summary, the current study specifically points out the possibility of an exposure of humans to the causative agents of cryptococcosis in house dust in the Rio Negro micro-region of the Brazilian Amazon, where the main genotypes described worldwide are present and show a high virulence. Our findings also point towards the need for a thorough investigation of the importance of the presence of highly virulent disease agents in indoor dust microenvironments. Future studies are now warranted to analyze the impact of early and continuous exposure to indoor dust on the development of subclinical or clinical cryptococcal infections amongst the inhabitants of those houses and to illustrate the impact of interaction between humans, animals and the environment as part of the one health concept and the implications of this for the development of cryptococcosis.

## Figures and Tables

**Table 1:** Data of the cryptococcal isolation from indoor dust collected from the Rio Negro micro-region of the Brazilian Amazon, and molecular characterization (*URA5*-RFLP and MLST types) of the positive environmental samples.

Cities	Collected Samples	Positive Samples	Frequency of Positivity (%)	Range of CFU/g *	Molecular Type Isolated	MLST Profiles
Santa Isabel do Rio Negro	51	3	5.9	2.500 – >50.000	VGII	ST20 (VGIIa), ST7 (VGIIb), ST5 and new STs 264, 266-268 (VGII)
Barcelos	12	2	16.7	600 – 1.300	VNI	ST5 and ST93 (VNI)
Novo Airão	16	2	12.5	200 – 300	VGII VNI	ST7 (VGIIb) and new ST445 (VGII) ST5 (VNI)
All cities	79	7	8.9	200 –>50.00	VNI and VGII	All above mentioned

* CFU/g: colony-forming unit per gram of dust; NA: not applicable.

**Table 2:** Median survival times (MST) of *Galleria mellonella* larvae after being inoculated with the different strains of cryptococcal isolates recovered in this study. Median survival time from *Cryptococcus neoformans* isolates (n=9) and *C. gattii* isolates (n=16) were compared with the highly virulent strain CDCR265 (VGIIa) to determine the degree of virulence. Strains that were of comparable virulence with CDRC265 (*p* > 0.05) are highlighted in grey.

Species	Strain	Place of Isolation	Mating Type	ST°	Number of Deaths	Median Survival Time (h)	*p*-Value	Virulence*
*C. neoformans*	ARA-P15-3	Novo Airão	alfa	5	8	192	0.0040	+
	BAR10-07	Barcelos	alfa	93	9	180	0.0374	+
	BAR10-13	Barcelos	alfa	93	10	144	0.0340	+
	BAR10-16	Barcelos	alfa	93	10	144	0.1089	++
	BAR10-19	Barcelos	alfa	93	10	144	0.9461	++
	BAR10-21	Barcelos	alfa	93	10	180	0.0065	+
	BAR08-1	Barcelos	alfa	93	10	156	0.1050	++
	BAR08-4	Barcelos	alfa	5	10	168	0.0320	+
	BAR08-17	Barcelos	alfa	5	9	156	0.0477	+
*C. gattii*	DW650-1	Santa Isabel do Rio Negro	alfa	7	9	240	<0.0001	+
	DW650-2	Santa Isabel do Rio Negro	alfa	5	7	192	<0.0001	+
	DW650-3	Santa Isabel do Rio Negro	alfa	264	8	204	<0.0001	+
	DW650-4	Santa Isabel do Rio Negro	alfa	264	7	192	0.0067	+
	DW650-5	Santa Isabel do Rio Negro	alfa	7	7	216	0.0002	+
	DW650-14	Santa Isabel do Rio Negro	alfa	7	10	144	0.5853	++
	DW650-24	Santa Isabel do Rio Negro	alfa	7	10	168	0.0041	+
	SI443-13	Santa Isabel do Rio Negro	alfa	266	10	132	0.6349	++
	SI443-14	Santa Isabel do Rio Negro	alfa	20	10	144	0.8420	++
	SI443-15	Santa Isabel do Rio Negro	alfa	20	10	156	0.1337	++
	SI443-17	Santa Isabel do Rio Negro	a	268	9	180	0.0097	+
	SI443-24	Santa Isabel do Rio Negro	alfa	267	9	156	0.0880	++
	SI444-1	Santa Isabel do Rio Negro	alfa	7	9	192	0.0004	+
	ARA-P9A	Novo Airão	alfa	445	8	204	<0.0001	+
	ARA-P15-1	Novo Airão	alfa	445	5	228	0.0621	+
	ARA-P15-2	Novo Airão	alfa	7	10	192	0.0005	+
Reference strain	CDCR265	Canada	alfa	20	10	132	NA	++

° Sequence type (ST); *(+) strains that killed at least one larva during the time of the experiment and were less virulent than the highly virulent Vancouver Island outbreak VGIIa strain CDRC265 (*p* < 0.05) and (++) strains that were of comparable virulence as CDRC265 (*p* > 0.05).
